# Ovarian Ecdysteroidogenesis in Both Immature and Mature Stages of an Acari, *Ornithodoros moubata*

**DOI:** 10.1371/journal.pone.0124953

**Published:** 2015-04-27

**Authors:** Mari Horigane Ogihara, Juri Hikiba, Yutaka Suzuki, DeMar Taylor, Hiroshi Kataoka

**Affiliations:** 1 Graduate School of Frontier Sciences, The University of Tokyo, Kashiwa, Chiba, Japan; 2 Faculty of Life and Environmental Sciences, University of Tsukuba, Tsukuba, Ibaraki, Japan; Onderstepoort Veterinary Institute, SOUTH AFRICA

## Abstract

Ecdysteroidogenesis is essential for arthropod development and reproduction. Although the importance of ecdysteroids has been demonstrated, there is little information on the sites and enzymes for synthesis of ecdysteroids from Chelicerates. Ecdysteroid functions have been well studied in the soft tick *Ornithodoros moubata*, making this species an excellent candidate for elucidating ecdysteroidogenesis in Chelicerates. Results showed that *O*. *moubata* has at least two ecdysteroidogenic enzymes, Spook (OmSpo) and Shade (OmShd). RNAi showed both enzymes were required for ecdysteroidogenesis. Enzymatic assays demonstrated OmShd has the conserved functions of ecdysone 20-hydroxylase. *OmSpo* showed specific expression in the ovaries of final nymphal and adult stages, indicating *O*. *moubata* utilizes the ovary as an ecdysteroidogenic tissue instead of specific tissues as seen in other arthropods. On the other hand, *OmShd* expression was observed in various tissues including the midgut, indicating functional ecdysteroids can be produced in these tissues. In nymphal stages, expression of both *OmSpo* and *OmShd* peaked before molting corresponding with high ecdysteroid titers in the hemolymph. In fed adult females, *OmSpo* expression peaked at 8–10 days after engorgement, while *OmShd* expression peaked immediately after engorgement. Mated females showed more frequent surges of *OmShd* than virgin females. These results indicate that the regulation of synthesis of ecdysteroids differs in nymphs and adult females, and mating modifies adult female ecdysteroidogenesis. This is the first report to focus on synthesis of ecdysteroids in ticks and provides essential knowledge for understanding the evolution of ecdysteroidogenesis in arthropods.

## Introduction

Molting is a common phenomenon in the development of arthropods because they must shed the exoskeleton to grow. This phenomenon is regulated by steroid hormones, namely ecdysteroids. In insects and crustaceans, ecdysteroids are commonly synthesized in a tissue specific for ecdysteroid synthesis [1]. Insects contain prothoracic glands (PGs), a pair of glands located near the prothoracic spiracle, as the organ for ecdysteroidogenesis. Although PGs degenerate by programmed cell death during metamorphosis, they are the obligate tissue for ecdysteroidogenesis in immature stages. Crustaceans also contain an ecdysteroidogenic tissue called the Y-organ located on the eye stalks [2]. In insects, production of ecdysone, a precursor of the functional ecdysteroid of most insects, occurs by processes involving at least 6 enzymes; Neverland, Non-molting glossy (Nm-g), CYP307A1 (Spook, Spo), CYP306A1 (Phantom), CYP302A1 (Disembodied) and CYP315A1 (Shadow) [3]. The production of ecdysone is initiated with the conversion of cholesterol into 7-dehydrocholesterol (7dC) by Neverland. 7dC is converted into another intermediate ketodiol through several unknown steps called a black box [3]. Genetic analysis showed Nm-g and Spo are required for reactions in the black box [4–6]. The reactions to synthesize ecdysone for ketodiol and combinations of steroid substrates and enzymes have been elucidated in insects [4–6]. All enzymes are localized in the PGs of insects allowing them to produce ecdysone. Synthesized ecdysone is secreted into the hemolymph and converted into a functional ecdysteroid, 20-hydroxyecdysone (20E), in various tissues by another ecdysteroidogenic enzyme, CYP314A1 (Shade, Shd), which hydroxylates ecdysone at the C-20 position [3]. A complex of 20E and its functional receptor, consisting of an ecdysone receptor and a retinoid X receptor (ultraspiracle in insects), regulates the transcription of genes required for molting. The complex of 20E and the functional receptor is also required for other ecdysteroid-dependent processes such as egg production [7, 8]. The existence of ecdysteroids and the receptor complex have been widely confirmed in arthropods [9], so the processes for ecdysteroidogenesis are expected to also be conserved in arthropods other than insects. In fact, a study with *Daphnia pulex* demonstrated that this crustacean also contains a set of ecdysteroidogenic enzymes, including *nvd*, *spo*, *phm*, *dib*, *sad* and *shd* [10].

In blood feeding acari, ecdysteroidogenesis is thought to be stimulated by engorgement. Nymphs of the soft tick *Ornithodoros moubata* showed increases in ecdysteroids in the hemolymph just prior to molting after a single engorgement [11]. This indicates nutrients act as a trigger for ecdysteroidogenesis in the nymphal stages. Ecdysteroids are also required for reproduction in ticks. Mated females produce much higher titers of ecdysteroids in the hemolymph than virgin females [11] indicating mating is necessary for adult females of *O*. *moubata* to complete ecdysteroidogenesis. Mated females also show high titers of ecdysteroids in the ovary that may be required for embryonic development. Engorgement and mating appear to be the key factors to stimulate ecdysteroidogenesis in *O*. *moubata*, but the molecular mechanisms and sites of ecdysteroidogenesis remain unknown. Synthesis of ecdysteroids in the epidermis has been reported from other ticks [12, 13], but a specific tissue for ecdysteroidogenesis has not been clearly elucidated. To understand ecdysteroidogenesis in *O*. *moubata*, identification of the ecdysteroidogenic enzymes was performed. In this study, we identified the genes encoding Spo and Shd from *O*. *moubata* and showed these genes were associated with ecdysteroidogenesis. Through expression analysis, the sites related to ecdysone and 20E synthesis were determined as the ovary and various tissues, respectively. This is the first report on the functional analysis of ecdysteroidogenesis in Chelicerates and contributes to understanding the evolution of ecdysteroidogenesis in arthropods.

## Materials and Methods

### Ticks

The soft tick *Ornithodoros moubata* was reared at 30±1°C, 70±10% RH in total darkness as described by Horigane et al. [14]. Fifth (final) instar nymphs or adult females were used in this assay. Final instar nymphs of this tick species normally molt into adult females after engorgement. Ticks were fed on rabbits as described by Chinzei et al. [15]. Rabbits were used in a humane manner after receiving approval from the Institutional Animal Experiment Committee of the University of Tsukuba, and in accordance with the Regulation for Animal Experiments at the university and Fundamental Guideline for Proper Conduct of Animal Experiment and Related Activities in Academic Research Institutions under the jurisdiction of the Ministry of Education, Culture, Sports, Science and Technology.

### RNA-seq

For the RNA-seq, total RNA of *O*. *moubata* was extracted from the whole body of final instar nymphs 1 or 9 days after engorgement. Cuticle of ticks was cut and exposed midguts punctured for removal of host blood from the tissues. Total RNA was extracted with TRIZOL reagent (Invitrogen). RNA preparation and sequencing were performed as described in Kanematsu et al. [16] and Iga et al. [17]. Sequences obtained by RNA-seq were deposited in DNA Data Bank of Japan Sequence Read Archive (Accession No. DRA002863). These sequences were assembled for contig construction using ABySS ver. 1.2.6 (http://www.bcgsc.ca/platform/bioinfo/software/abyss) and the homology analysis for annotation was performed using tblastx.

### First strand cDNAs for rapid amplification of cDNA ends (RACE)

cDNAs for RACE were synthesized by SMARTer RACE cDNA Amplification Kit (Clontech) with total RNA extracted from final instar nymphs. To obtain 5’ or 3’ ends of *OmSpo* and *OmShd*, PCRs were performed with RACE cDNA using Ex *Taq*, Ex *Taq* HS or LA *Taq* (TaKaRa Bio). All primers used in this study are presented in Table 1.

**Table 1:** Primers used in this study.

Experiments	Primer	Sequences
Spo Cloning	F1	CTATGGACGATGGAGCACCT
	F2	AACTGTGTCTGGGCTCTCGT
	F3	GTCTGCATATCTAGGCTTACTACAGAGATA
	R1	CGTCAGCCACATCTTTCTCA
	R2	AGATAGCATGATAGCGAAGAAAGTCT
	R3	CGTCAGCCACATCTTTCTCA
	R4	TCTTGAAAGACGGCATCGTAGAGTC
	R5	AGATAGCATGATAGCGAAGAAAGTCT
Shd Cloning	F1	GCAGATGAAGAACGTACAGACACT
	F2	CACAGCAAAAGTAAATTGGCTCTAC
	F3	ACTGTGCTCCCAACAGGAAC
	F4	AATAATGGATTTTATAGCAGGAGGAGT
	R1	CGAGATACCTCAGCGTGACA
	R2	GGTCTTGAATGTTGTCGATAGAATC
	R3	CACTTTCCCCCACATCATTT
	R4	GGAGGTCGTCACGGAATGTCT
p IZT	F_Spe	GTGTGTACTAGTATGAGGCAGAGCTGCGGGCGGCTCGT
	R_NotI	GTGTGTGCGGCCGCCGGAGGTCGTCACGGAATGTCT
dsRNA/WISH	SpoF	AACTGTGTCTGGGCTCTCGT
	SpoR	AGGTGCTCCATCGTCCATAG
dsRNA	ShdF	TCGGAACACTTTGCGATGTA
	ShdR	ACTGTAGGGCTGAGCCTGAA
dsRNA	GFPF	ATGAGTAAAGGAGAAGAACTTTT
	GFPR	TTTGTATAGTTCATCCATGCCA
qRT-PCR	ActF	ATGGTGGGTATGGGTCAGAA
	ActR	AGGTGTGGTGCCAGATCTTC
	Spo_RealF	TTCTCGGAAAGACTGCCAAT
	Spo_RealR	GAGGGTGCAGCCAACTATGT
	Shd_RealF	ACCGGTTCACTCTGGAATCA
	Shd_RealR	GCCCATAGTACAGCCTCTGG

Fragments obtained were purified using a Wizard SV Gel and PCR Clean-up System (Promega), subcloned into a pGEM-T Easy Vector (Promega) and transformed into *E*. *coli* DH5α. Plasmids were extracted using FastGene Plasmid Mini Kit (Nippon Genetics). Sequencing reactions were performed using Big Dye Terminator v3.1 Cycle Sequencing Kit (Applied Biosystems) and sequences of *OmSpo* and *OmShd* determined using 3130xl and 3500 Genetic Analyzers (Applied Biosystems). Full OmSpo and OmShd sequences were deposited to DNA Data Bank of Japan (Accession No. of OmSpo: LC017702, Accession No. of OmShd; LC017701).

### Genetic and phylogenetic analyses of OmSpo and OmShd

Homology analyses of amino acid sequences for OmSpo and OmShd were performed with EMBOSS Water Program (http://www.ebi.ac.uk/Tools/psa/emboss_water/). For the construction of a phylogenetic tree, the amino acid sequences of Spo, Phm, Dib, Sad and Shd orthologs were obtained from GenBank (http://www.ncbi.nlm.nih.gov/genbank/). Multiple alignments of these P450s were performed by Clustal X2 program [18]. After multiple alignment, phylogenetic trees were constructed by the Neighbor-joining method [19]. Bootstrap values were assessed at 1000 replicates.

Gene numbers for Spo used for this study were *O*. *moubata* (this study, LC017702), *B*. *mori* (BAH47267), *Daphnia pulex* (EFX88041), *D*. *melanogaster* (NP_001104460.2), *Ixodes scapularis* (XP_002401790), *Metaseiulus occidentalis* (XP_003747195), *Pediculus humanus corporis* (XP_002429996) and *Tribolium castaneum* (XM_969184). Gene numbers of Shd were *O*. *moubata* (this study, LC017701), *B*. *mori* (AB236417), *Daphnia pulex* (EFX77008), *D*. *magna* (BAF35770), *D*. *melanogaster* (AF484414), *Ixodes scapularis* (XP_002401077), *Metaseiulus occidentalis* (XP_003748577), *Pediculus humanus corporis* (XP_002425642) and *T*. *castaneum* (NM_001130422). Numbers of other genes were *B*. *mori* Phm (BAD34476), *B*. *mori* Dib (AB124841), *B*. *mori* Sad (AY947551), *D*. *melanogaster* Phm (AF484413), *D*. *melanogaster* Dib (NM_139718), *D*. *melanogaster* Sad (AY079170), *T*. *castaneum* Phm (XP_968477), *T*. *castaneum* Dib (XM_969159) and *T*. *castaneum* Sad (XM_965029). The amino acid sequence of CYP1A1 for an outgroup was *Homo sapiens* (AAH23019).

### Enzymatic assay of OmShd by LC-MS/MS

To construct an expression vector of OmShd (pIZT-OmShd), full length *OmShd* was amplified by KOD FX Neo (Toyobo) and introduced in to a modified pIZT/V5-His vector (Invitrogen), this added a single HA tag. The empty vector (pIZT) was used as the negative control. pIZT-OmShd or pIZT vector (250 μg) was transfected to *Drosophila* S2 cells with Lipofectamin LTX (Life Technologies). After 24 h transfection, 100 ng of ecdysone (Sigma Aldrich) was added to each sample. S2 cells were cultured at 25°C for the experiments. Steroids were extracted as described in Hikiba et al. [20]. Steroids were resolved in methanol and separated on HPLC with a Develosil C30-UG column (φ2.0 mm x 50 mm, Nomura Chemical) and 25–100% linear gradient of acetonitrile (flow rate of 0.4 ml/min). Steroids were analyzed with QTRAP5500 (Applied Biosystems). Standard ecdysone and 20-hydroxyecdysone (20E) used in this study were purchased from Sigma Aldrich.

### Quantitative RT-PCR (qRT-PCR)

Total RNA was extracted with TRIZOL reagent (Invitrogen). cDNA syntheses were performed with PrimeScript RT Reagent Kit (Perfect Real Time) (TaKaRa Bio). To determine the expression site, cDNA was prepared from synganglions (tick brain), salivary glands, ovaries, reproductive tracts (uterus and oviducts), midguts, fat bodies and the remaining tissues (mainly cuticle, muscles and a small amount of attached fat body) of final instar nymphs 9 days after engorgement and mated females 2 days after engorgement. Ticks have a single large gut corresponding to the midgut of insects and the whole gut was used for expression analyses. Tick fat body is a very fine tissue associated with the trachea [14], so fat bodies were collected with the trachea. To determine developmental profiling of *OmSpo* and *OmShd* expression, ovaries and midguts were used, respectively. *Actin* was used as an internal control [21]. Absolute quantification of *OmSpo*, *OmShd* and *OmAct* were performed with SYBR Premix Ex Taq II (T li RNase plus, TaKaRa Bio) on a Thermal Cycler Dice Real Time System (TaKaRa Bio). Final concentrations of all primer sets were 0.05 μM and PCR conditions were as recommended by the manufacturer. PCR reactions were performed for 30 s at 95°C, and 40 cycles at 95°C for 5 s and 60°C for 60 s. Three to four individual ticks were used for each day.

### Knockdown of OmSpo and OmShd by RNAi

A fragment of *OmSpo* or *OmShd* amplified by Ex*Taq* (TaKaRa Bio) was introduced into a pGEM-T Easy Vector (Promega) and utilized as a template to synthesize double strand RNA (dsRNA). As a negative control, GFP was amplified from the plasmid UAS-GFP.RN3 [22] which contained GFP and subcloned into a pGEM-T Easy vector. Plasmids containing fragments were linearized with Spe I (TaKaRa Bio) or Nco I (TaKaRa Bio) and RNA polymerase reactions were performed using T7 RNA polymerase (TaKaRa Bio) or SP6 RNA polymerase (Invitrogen), respectively. Single strand RNAs were mixed and heated at 75°C for 5 min and cooled gradually for annealing as dsRNA. dsRNA obtained was purified by RQ DNase I (Promega) and RNAase A (Sigma Aldrich). After phenol precipitation, dsRNA was resolved in PBS (1 μg dsRNA/ 2 μl PBS) and stored at -20°C until analysis. Two microliters of dsRNA (1 μg dsRNA) were injected into the nymphs 2 days after engorgement using a glass capillary. Inhibition of gene expression by dsRNA injection was confirmed 1, 3 and 7 days after injection (3, 5, and 9 days after engorgement) by qRT-PCR. The statistical significance of the expression levels in nymphs injected with dsRNA was evaluated by a Welch t-test.

For rescue of dsSpo injected nymphs, 1 μg of 20E (Sigma Aldrich) was injected 7 days after dsSpo treatment (9 days after engorgement). Development and eclosion of nymphs after dsRNA injection were observed daily. The statistical significance of days after ecdysis in the nymphs injected with dsRNA was evaluated by a Welch t-test.

### Whole mount *in situ* hybridization (WISH)

WISH was modified based on Horigane et al. [14]. The plasmids for dsRNA synthesis were the same as used to synthesize dsSpo. Sense and antisense RNA probes were synthesized using T7 RNA polymerase (TaKaRa Bio) or SP6 RNA polymerase (Invitrogen) and PCR DIG labeling mix (Roche Diagnostics). Anti-Digoxigenin fragment AP (Roche Diagnostics), Nitro-blue tetrazolium (NBT, Promega) and 5-bromo-4-chloro-3’-indolyphosphate (BCIP, Promega) were used for detection. Localization of *OmSpo* transcripts was determined with final instar nymphs 9 days after engorgement or mated females 2 days after engorgement.

## Results

### Identification of ecdysteroidogenic enzymes from *Ornithodoros moubata*

To identify ecdysteroidogenic enzymes, sequences annotated as cytochrome P450s (CYPs) were surveyed. RNA-seq data was established with *de novo* assembly of sequence data from final instar nymphs 1 or 9 days after engorgement, corresponding with the periods for low and high ecdysteroid titers in the hemolymph [11]. Contigs for CYP307A1 (OmSpo) homolog were found in the RNA-seq data of both 1 and 9 days after engorgement, while a contig for CYP314A1 (OmShd) was found only in the data produced by nymphs 1 day after engorgement. There were several CYP contigs in the RNA-seq data (data not shown), but homologs of other CYPs for ecdysteroidogenesis were not found.

**Fig 1:**
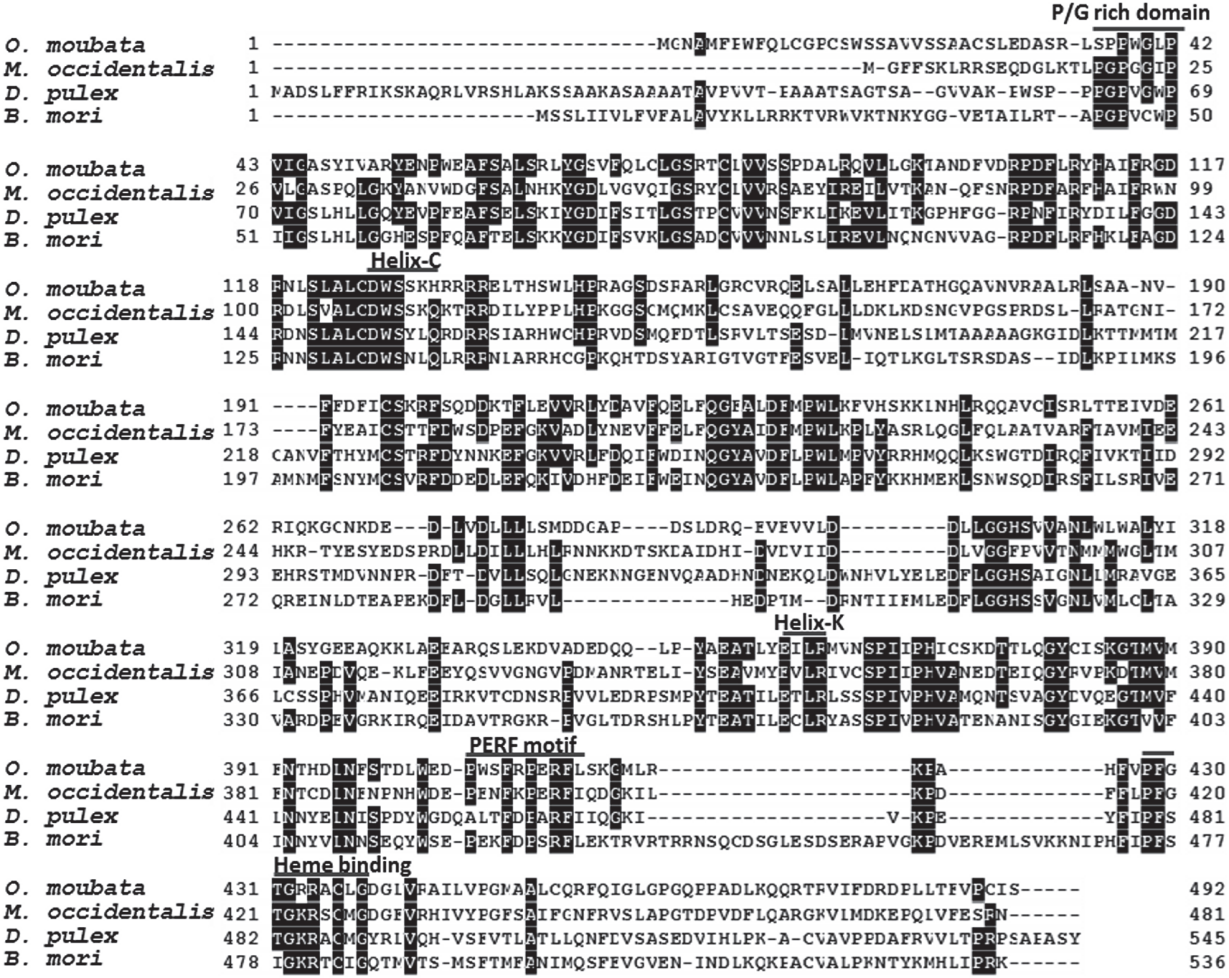
Multiple alignment of OmSpo with orthologs determined for other arthropods. Location of a P/G rich domain, Helixes (Helix-C and Helix-K), a PERF motif and a Heme binding domain are indicated

**Fig 2:**
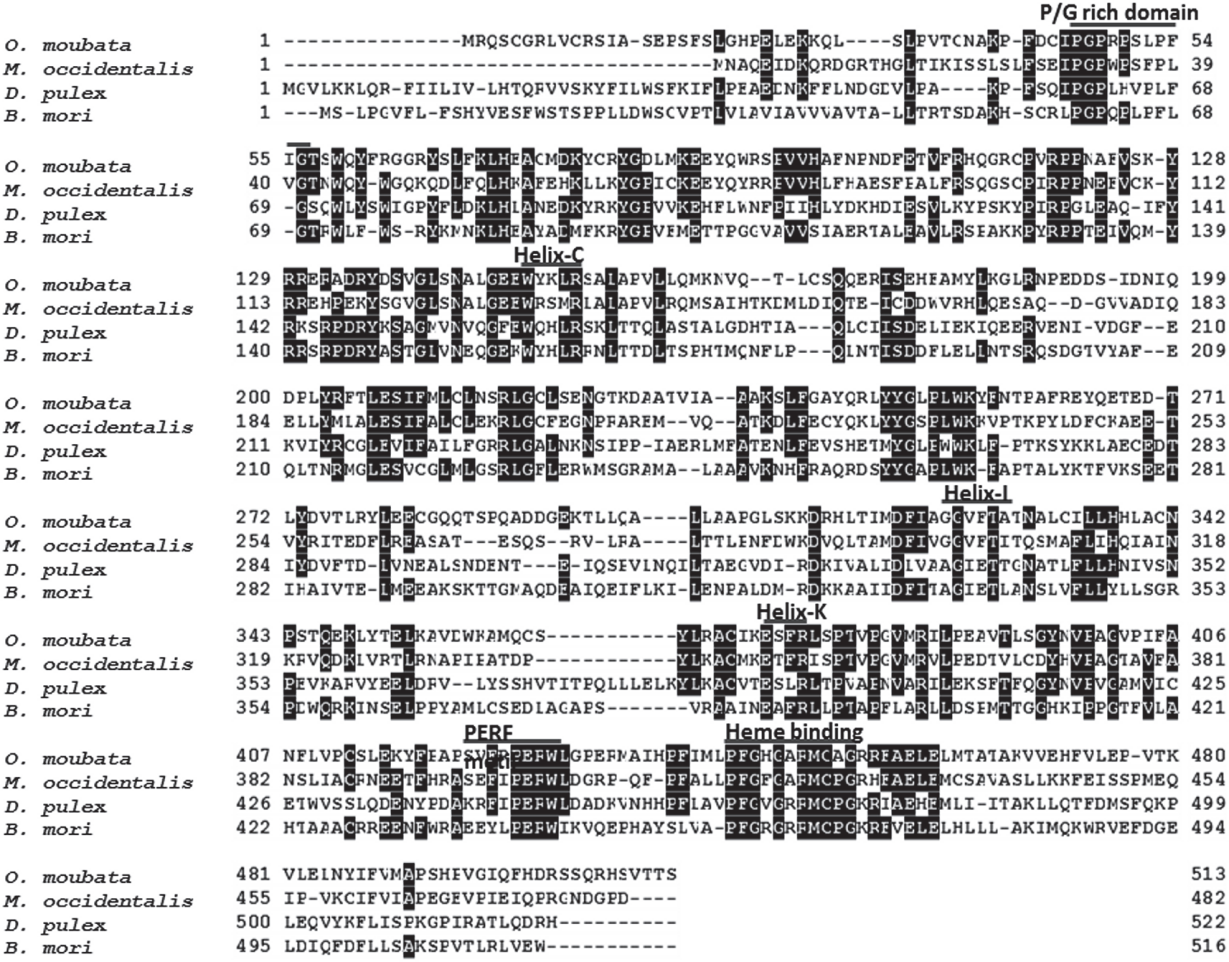
Multiple alignment of OmShd with orthologs determined for other arthropods. Location of a P/G rich domain, Helixes (Helix-C, Helix-I and Helix-K), a PEFR motif and a Heme binding domain are indicated.

**Fig 3:**
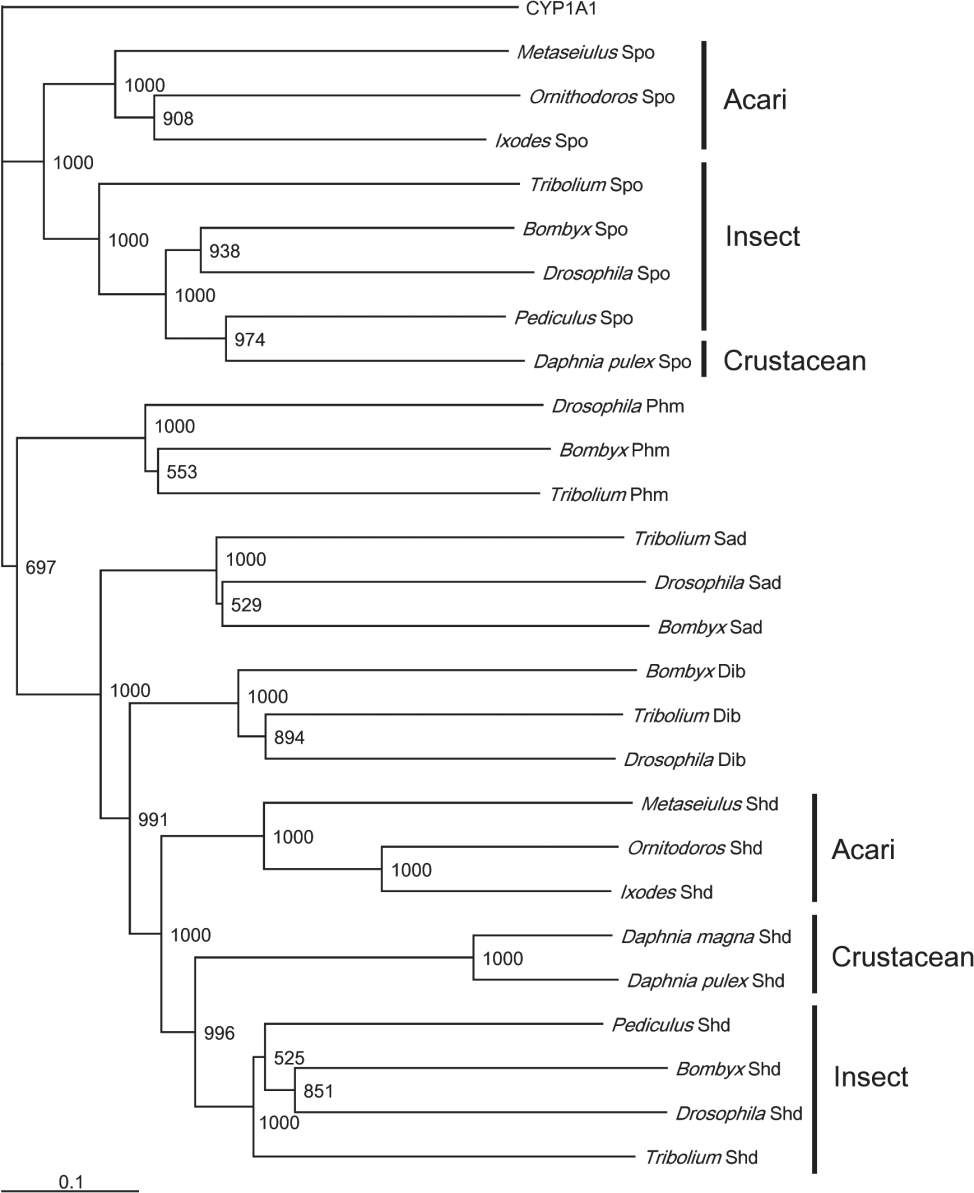
Phylogenic tree of CYPs associated with ecdysteroidogenesis. A phylogenetic tree of CYPs was constructed by the Neighbor-joining method. The bootstrap values (1000 replications) are represented at the branches. The scale bar indicates the numbers of amino acid substitutions per site. Gene numbers used for this analysis are described in the materials and methods.

Full sequences of *OmSpo* and *OmShd* were determined using 5’ and 3’ RACE (S1 and S2 Figs). Sequences showed conserved motifs: P/G rich regions, helices, PERF motifs and heme binding domains (Figs 1 and 2). These sequences showed the highest homologies with the orthologs of a hard tick *I*. *scapularis*. As well as the orthologs from *I*. *scapularis*, OmSpo and OmShd shared high homologies (40–60% similarities) with other orthologs (Table 2). Phylogenetic analysis showed the sequences obtained were classified into clades of the respective orthologs (Fig 3). These results indicate the sequences are orthologs of Spo or Shd. In addition to *O*. *moubata*, orthologs of Spo and Shd were found in the genomic database of *I*. *scapularis* (hard tick) indicating processes related to these enzymes are conserved in ticks.

**Table 2:** Homologies of OmSpo and OmShd to their orthologs in other arthropods.

Classification	Species	Spook		Shade	
		Identity (%)	Similarity (%)	Identity (%)	Similarity (%)
Acari	*Ixodes scapularis*	49.4	72.2	64.6	75.4
	*Metaseiulus occidentalis*	43.2	65.3	44.2	60.3
Crustacean	*Daphnia pulex*	35.4	55.1	34.1	52.0
Insect	*Acyrthosiphon pisum*	33.1	52.4	44.2	60.3
	*Bombyx mori*	34.1	52.7	34.7	51.0
	*Drosophila melanogaster*	30.0	49.1	33.6	52.7

### Functional analysis of Spo and Shd of *O*. *moubata*

Although the primary structures of OmSpo and OmShd showed common characteristics with their orthologs, variations were observed in the conserved motifs (Figs 1 and 2). To confirm whether OmSpo and OmShd are associated with ecdysteroidogenesis, gene suppressions using RNAi were performed. Nymphs injected with dsGFP (negative control) molted normally approximately 11 days after engorgement (11.1±1.4 days) (Table 3). Nymphs injected with dsSpo showed no development, such as apolysis, for pharate nymphs and no molting (Table 3) with suppressed *OmSpo* expression at least 3 and 7 days after injection (S3 Fig). Nymphs injected with dsShd showed delayed molting and abnormal ecdysis compared to the controls (Table 3 and S3 Fig), even though the reduction of *OmShd* expression was not significant and did not continue (S3 Fig). The exuvia of nymphs injected with dsShd showed abnormal white dapples (S3 Fig), unlike the light orange-colored exuvia of control nymphs. Therefore, reduction of *OmSpo* induced arrest of development, whereas reduction of *OmShd* induced abnormalities in molting.

**Table 3:** Effects of dsRNA injection on ecdysis of final instar nymphs.

dsRNA	Numbers of adults	Days for ecdysis	Numbers of abnormal ecdysis
dsGFP	54/56	11.1 (±1.4)	0/56
dsSpo	0 /56	-	-
dsShd	50/51	12.3 (±2.0)*	48/51

Statistical significance (p<0.01) between nymphs injected with dsGFP and dsShd are represented with *.

The injection of 20E rescued the defective molting caused by dsSpo injection (Table 4). After 20E injection, all nymphs developed into pharate nymphs and more than half of the nymphs emerged, so OmSpo appears to produce ecdysteroids required for nymphal development.

**Table 4:** Effects of 20E injection of nymphs after dsSpo injection.

	Mock	20E+
Pharate	0/30	30/30
Emergence	0/30	16/30

**Fig 4:**
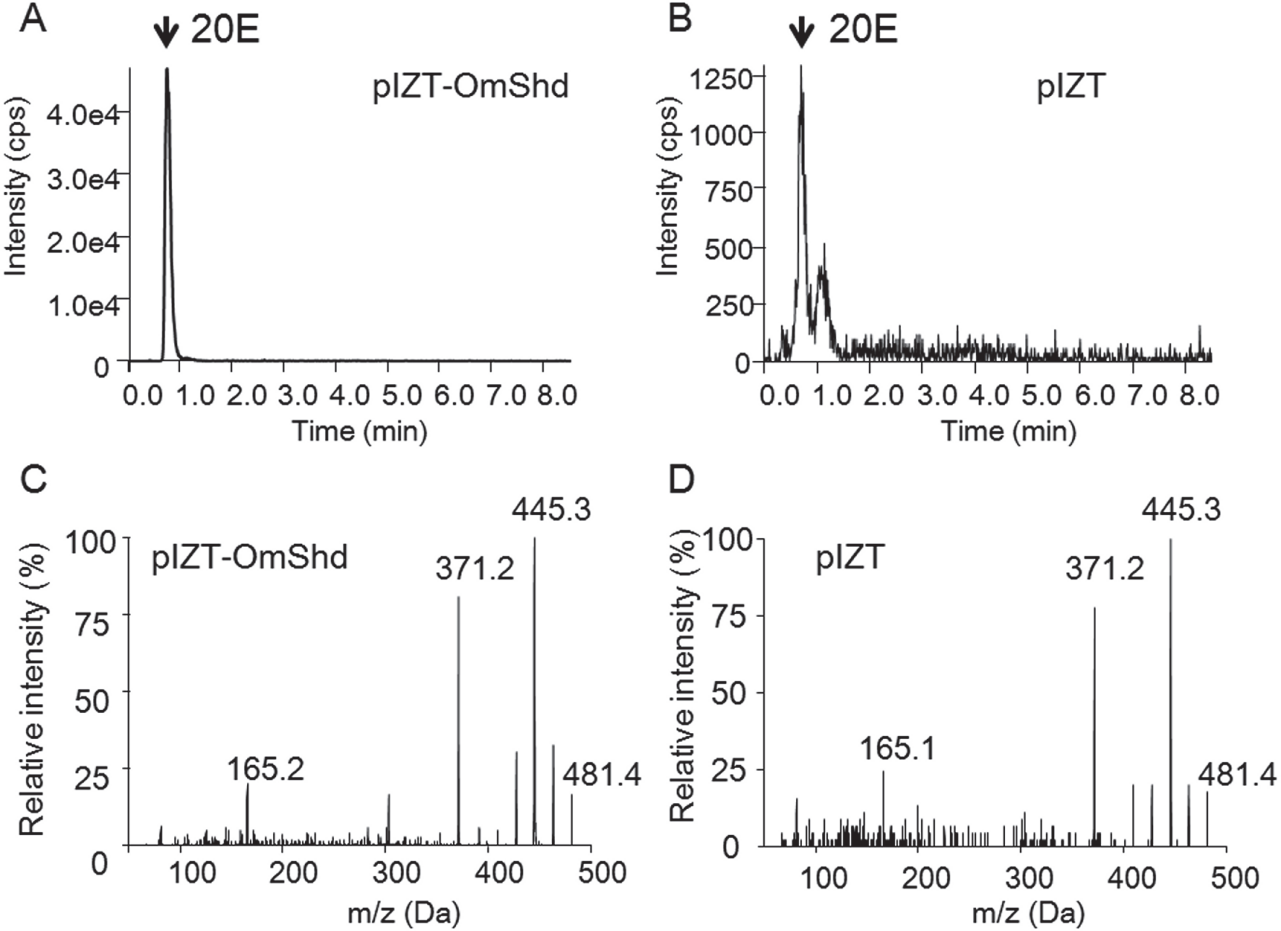
Chromatograms of 20-hydroxyecdysone (20E), produced by enzymatic assay of OmShd, analyzed with LC-MS/MS. 20E chromatograms from the medium of pIZT-OmShd vector (A) or pIZT vector (negative control) (B) were transfected to S2 cells. Intensity was indicated as counts per second (cps). MS/MS spectra of 20E standard (C) and 20E produced by OmShd (D).

Shd is an ecdysone 20-hydoroxylase, and this function of OmShd was confirmed *in vitro* (Fig 4). 20E was detected in the medium when S2 cells were transfected with OmShd, and cultured with ecdysone as a substrate (Fig 4A). Only a small peak of 20E was detected in the medium with S2 cells transfected with the pIZT vector (control) (Fig 4B). The 20E product converted from ecdysone by OmShd showed typical MS/MS spectra for 20E (Fig 4C and 4D), showing OmShd has ecdysone 20-hydroxylase activity.

Changes in ecdysteroid titers after dsRNA injection were investigated using LC-MS/MS (S4 Fig). Peaks that had the same retention times as ecdysone and 20E were detected in the hemolymph of nymphs injected with dsGFP and dsShd (S4 Fig). These materials showed the same MS/MS spectra as standard ecdysone and 20E (S4 Fig). Ponasterone A could not be detected in the hemolymph of all nymphs injected with dsRNA. Therefore, we conclude that *O*. *moubata* utilizes ecdysone as a precursor and 20E as a molting hormone. These ecdysteroids were not detected from the hemolymph of nymphs injected with dsSpo (S4 Fig). Knockdown of dsSpo appeared to inhibit production of ecdysone and 20E. In contrast to nymphs injected with dsSpo, ecdysone and 20E were detected in the hemolymph of nymphs injected with dsShd (S4 Fig). Because *OmShd* expression increased during the 7 days after dsShd injection (S3 Fig), nymphs injected with dsShd could produce 20E. On the other hand, the amount of ecdysone in the hemolymph of nymphs injected with dsShd was much higher than in the control nymphs (S4 Fig), indicating, suppression of OmShd allows for the accumulation of ecdysone by blocking utilization. These results show that OmShd is required for ecdysone conversion to 20E *in vivo*.

### Determination of the tissue for ecdysteroidogenesis

**Fig 5:**
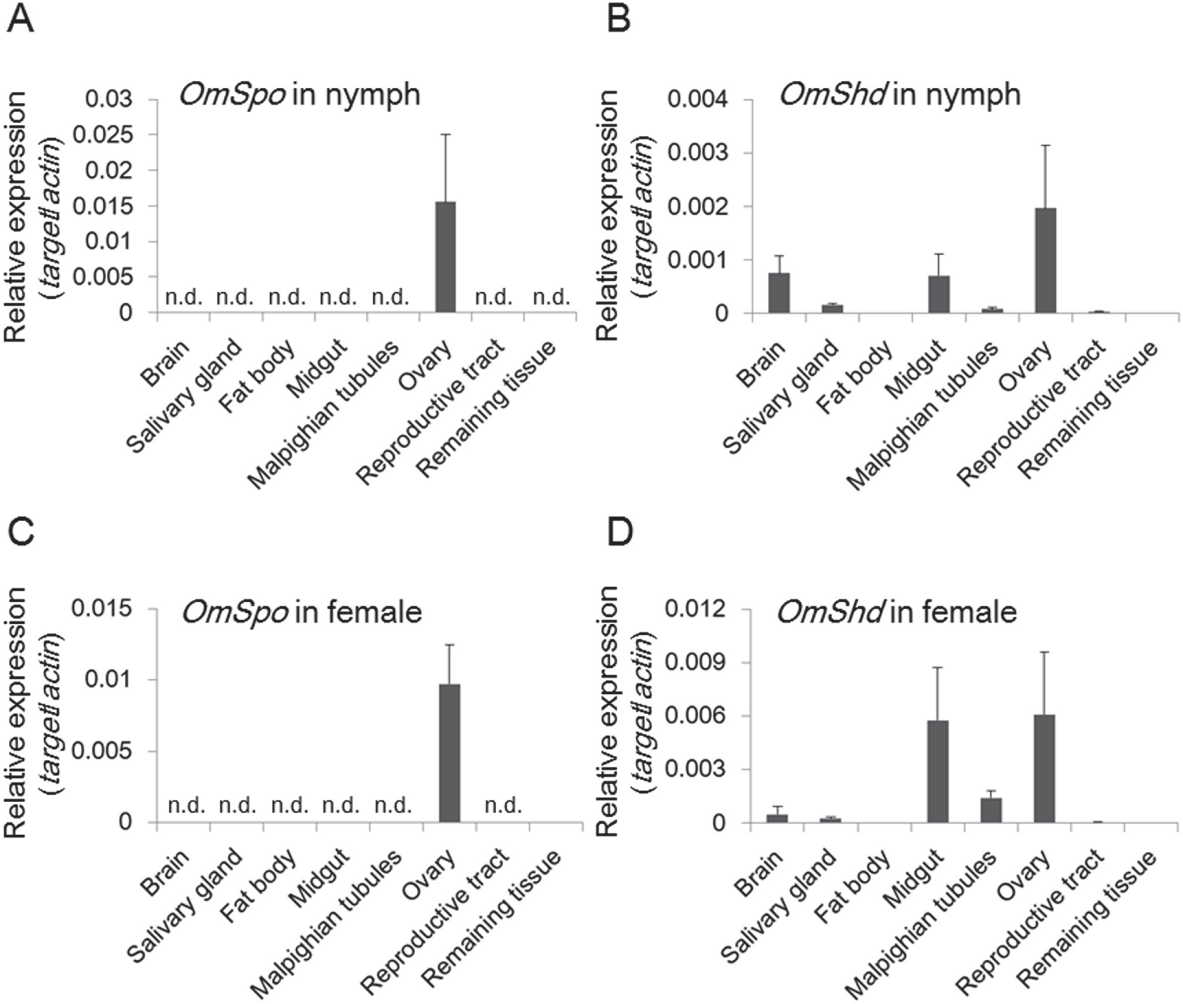
Expression sites of *OmSpo* and *OmShd* in nymphs and adult females of *O*. *moubata*. *OmSpo* (A) and *OmShd* (B) expression in final instar nymphs determined 9 days after engorgement by qRT-PCR (n = 3–4, mean±se). OmSpo (C) and OmShd (D) expression in mated females determined 2 days after engorgement (n = 3–4, mean±se). n. d. indicates no detection.

**Fig 6:**
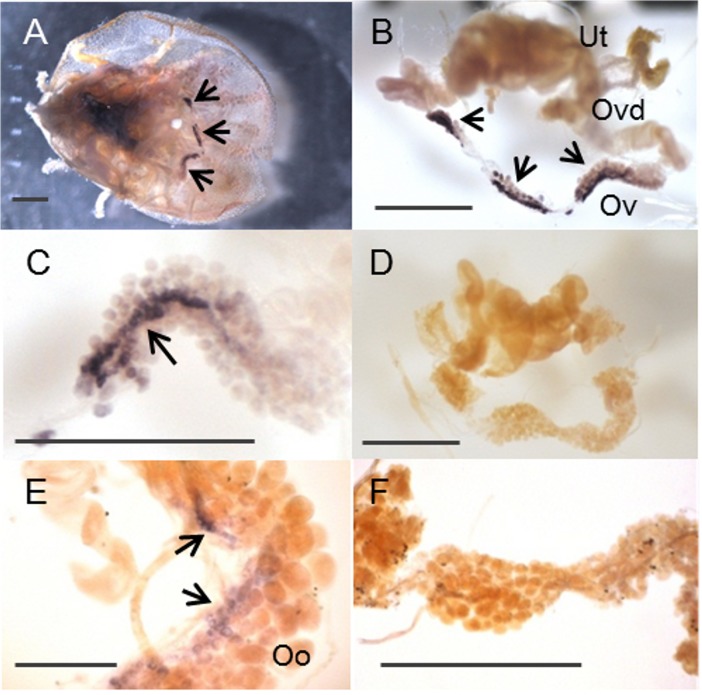
*OmSpo* expression in specific cells of ovaries from nymphs and adult females determined by WISH. An overall ventral view of tick tissues in a final instar nymph (A). *OmSpo* localization in dissected reproductive tissues from the nymph in Fig 6A (B). *OmSpo* localization in small cells of the ovary with a band-like configuration (C, magnification of Fig 6B). Ovary of a final instar nymph stained with the sense probe (D). *OmSpo* localization in the ovary of a mated female (E). Ovary of mated female stained with the sense probe (F). Arrows indicate staining of *OmSpo* with the antisense probe. Ticks have a single ovary (Ov) connected to oviducts (Ovd) from both sides. Oviducts open into a uterus (Ut). Developing oocytes (Oo) are attached outside the ovary. Scale bars indicate 1 mm.

Ticks have no prothoracic gland-like organs, so qRT-PCR was performed to determine the sites of *OmSpo* and *OmShd* expression (Fig 5). The expression patterns of *OmSpo* and *OmShd* were determined for synganglions (tick brains), salivary glands, fat bodies, midgut, Malpighian tubules, ovaries, reproductive tracts and the remaining tissues. Tick fat body is a very fine tissue mainly associated with trachea that elongate from spiracles. Reproductive tracts included oviducts and uterus. Remaining tissues included cuticle, fat bodies and muscles. Most final instar nymphs molt into adult females, and ovaries from these nymphs showed *OmSpo* expression (Fig 5A). No other tissues in the final instar nymphs showed *OmSpo* expression. Ovaries in fed adult females also showed *OmSpo* expression (Figs 5C and 6E). Low expression of *OmSpo* was detected in the remaining tissues of fed adult females (Fig 5C). Whole mount *in situ* hybridization (WISH) also showed *OmSpo* expression in the ovaries (Fig 6). Staining with sense probes detected no expression of *OmSpo* from ovaries of both nymphs and adult females (Fig 6D and 6F). *OmSpo* expression was detected in the small cells and band-like configurations in ovaries of both last instar nymphs and adult females, but not in the developing oocytes in adult females (Fig 6E). These cells in the ovary appear to be responsible for ecdysteroidogenesis in both nymphs and adult females.

Expression sites of *OmShd* were observed in various tissues such as the midgut and ovary, but only slight expression was detected in the fat bodies (Fig 5B and 5D). The synganglions of final instar nymphs also showed high *OmShd* expression, whereas mated females did not show such expression. Synganglion in nymphs just before ecdysis may require 20E as positive feedback to prepare for ecdysis. Synganglions of adult females may not require such stimulation. *OmShd* expressed in these tissues is expected to produce a functional ecdysteroid for the regulation of molting and egg production.

### Developmental changes in *OmSpo* and *OmShd* expression

**Fig 7:**
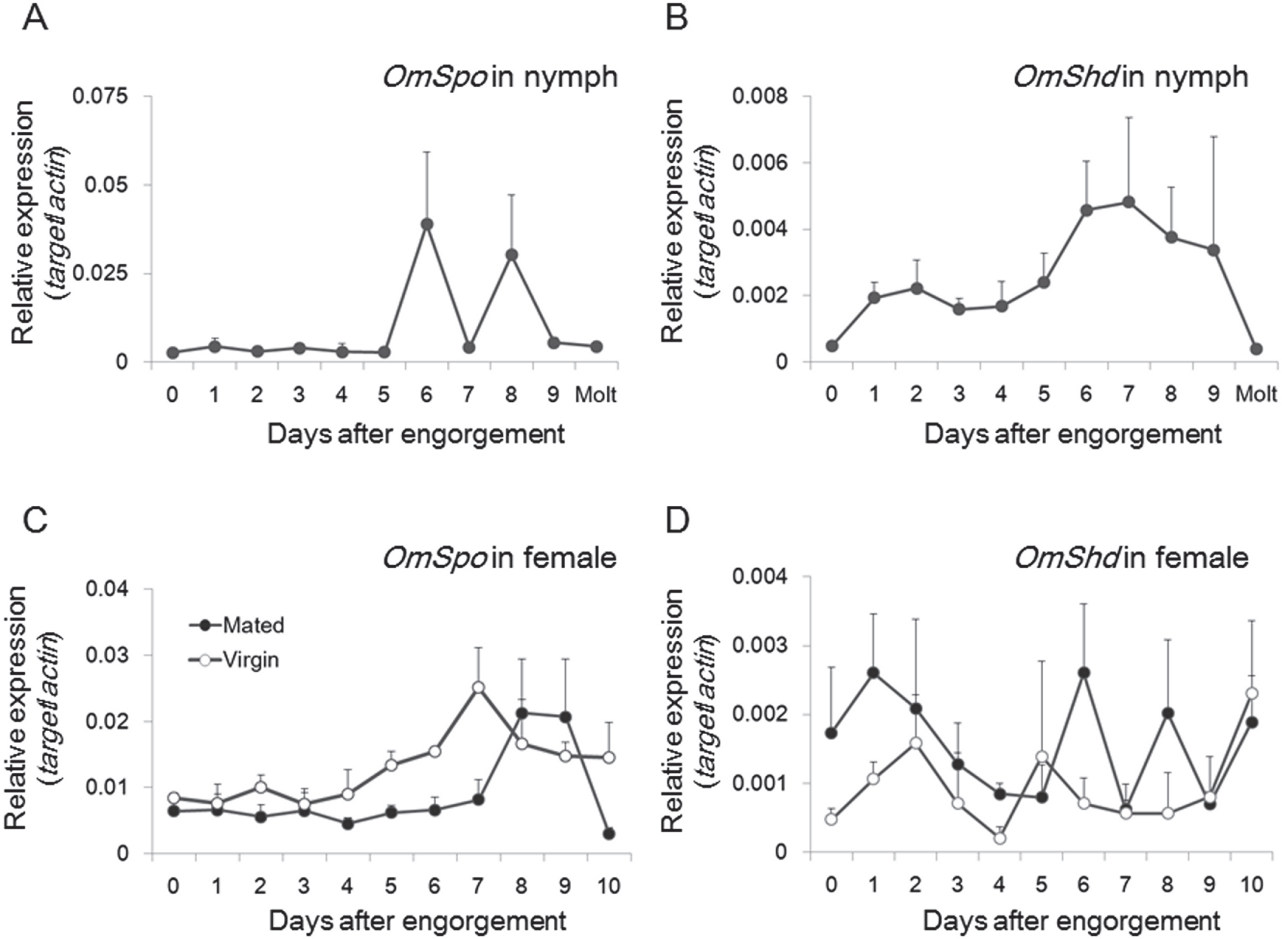
Developmental changes in *OmSpo* and *OmShd* expression during nymphal development or egg maturation in adult females. *OmSpo* (A) and *OmShd* (B) expression in nymphs was determined for ovaries and midgut, respectively. *OmSpo* (C) and *OmShd* (D) expression in adult females determined for ovaries and midgut, respectively. (n = 3–4, mean±se)

*OmSpo* and *OmShd* expression was determined with ovaries or midguts from both final instar nymphs and adult females after engorgement (Fig 7). Midgut is the largest tissue and expected to provide the highest contribution to 20E production. Therefore, expression analysis of *OmShd* was performed with the midgut. In nymphs, both *OmSpo* and *OmShd* expression gradually increased and peaked before molting (Fig 7A and 7B). Increases in expression of these genes can induce ecdysteroidogenesis for the regulation of molting.

In contrast to the nymphal stage, *OmSpo* and *OmShd* showed different expression patterns in adult females. In mated females, *OmSpo* expression gradually increased and peaked approximately 8–9 days after engorgement (Fig 7C), while *OmShd* showed several surges soon after engorgement (Fig 7D). Regulatory mechanisms for ecdysteroidogenesis may differ in nymphs and adult females. Comparison of mated and virgin adult females showed little differences in *OmSpo* expression. However, surges of *OmShd* expression were more frequent in mated adult females than in virgin adult females, indicating mated adult females produce a functional ecdysteroid that stimulates mature egg production.

## Discussion

### Identification of Spook and Shade from Acari

In this study, molecular mechanisms of ecdysteroidogenesis were examined in the acari *Ornithodoros moubata* to shed light on the synthesis of ecdysteroids in ticks. Two ecdysteroidogenic enzymes, Spook and Shade, that are necessary for ecdysteroidogenesis were identified from *O*. *moubata*.

To determine ecdysteroidogenic enzymes of *O*. *moubata*, RNA-seqs were performed and provided two candidate ecdysteroidogenic enzymes, Spook (OmSpo) and Shade (OmShd) (Figs 1 and 2). Other ecdysteroidogenic CYPs were not detected from *O*. *moubata* in this study. Genomic analyses showed that arthropods, including mites, have conserved ecdysteroidogenic enzymes [10, 23, 24], thus ticks may utilize similar enzymes. More detailed analysis based on whole genomic information of this tick and a transcriptome under different conditions is required to determine other ecdysteroidogenic enzymes.

Gene silencing by RNAi and enzymatic assays showed OmSpo and OmShd are associated with tick ecdysteroidogenesis. In this study, we clarified *O*. *moubata* has ecdysone and 20E, but not Ponasterone A (S4 Fig). Therefore, this tick likely utilizes 20E as a molting hormone. Nymphs injected with dsSpo had no peaks of ecdysone or 20E (S4 Fig). 20E is produced by Shd, so lack of these ecdysteroids indicates OmSpo is required for ecdysone synthesis. Spo is required for an early step in the black box of ecdysone synthesis [3], so OmSpo would likely be associated with this same step. Suppression of *OmSpo* resulted in developmental arrest with no molting (Table 3) and 20E injection rescued the developmental arrest (Table 4). Nymphs injected with dsShd showed accumulation of ecdysone (S4 Fig). *In vitro* enzymatic assays showed OmShd is ecdysone 20-hydroxylase (Fig 3). These results indicate OmShd is associated with 20E production. Nymphs injected with dsShd showed delayed molting and abnormal cuticle production (Table 3). In most arthropods, 20E regulates physiological changes for molting, including cuticle production [25]. Therefore, OmSpo is the enzyme involved in ecdysone synthesis and necessary for molting of this tick through the production of 20E.

Although the enzymatic functions of OmSpo and OmShd were similar in orthologs, the affinity of substrates may differ between orthologs. OmSpo and OmShd showed substitutions of amino acids in the conserved domains (Figs 1 and 2) and approximately 50–60% similarities with orthologs of insects and a crustacean, or 65 and 60% with a spider mite (Table 2). These differences may reflect different affinities for these enzymes. Actually, ecdysteroids other than ecdysone and 20E were detected, such as Ponasterone A in spider mites and 3-Dehydro-20-hydroxyecdysone in crustaceans [2, 23]. Comparisons of affinities between orthologs of ecdysteroidogenic enzymes and distinct ecdysteroid substrates will greatly help the understanding of the evolution of ecdysteroidogenic pathways in arthropods.

### Localization of *OmSpo* in the ovaries and *OmShd* in various tissues of nymphs and adult females

In insects, Spo is specifically localized in the ecdysteroidogenic tissue, PGs. We showed OmSpo is associated with ecdysone synthesis, so we determined the sites of *OmSpo* expression to clarify the ecdysteroidogenic tissue in *O*. *mobuata*. qRT-PCR and WISH showed *OmSpo* was mainly localized in the ovaries of both nymphs and adult females (Figs 5 and 6). Therefore, reactions catalyzed by OmSpo should occur mainly in the ovary. Limited *OmSpo* expression in the ovary of the final instar nymphal stage is different from insects, because Spo in insects is localized mainly in the PGs of immature stages [4, 6, 26]. *O*. *moubata* may utilize the ovary without developing a specific ecdysteroidogenic tissue or such a tissue may have been lost. *Spo* expression in several insects shifts to the ovary during oogenesis after PG degradation. The expression of *Spo* in adult ovaries has also been reported from the varroa mite [24]. Therefore, ovarian expression of ecdysteroidogenic enzymes in adult stages may be universal in arthropods.

In previous studies, ecdysone and 20E were detected in the epidermis and cultured media of the tissues of two different ticks [12, 13]. On the contrary, we showed the expression site of *OmSpo* is the ovary in *O*. *moubata*, indicating the ovary as the site for ecdysteroidogenesis. Possibly the subsequent steps occur in other tissues, including the epidermis, after conversion by OmSpo. Further studies to determine the expression sites of other ecdysteroidogenic enzymes and comparison of their expression in different species is required to elucidate the entire process of ecdysteroidogenesis in ticks. Studies on the tissues for ecdysteroidogenesis in males and younger instar nymphs also should help clarify the site of ecdysteroidogenesis in ticks.

*OmSpo* expression was detected as a band-like configuration in the smaller cells of ovaries, but not detected in developing oocytes of adult females (Fig 6). Insects show *Spo* expression in follicle and nurse cells in ovaries during egg maturation [4, 6]. Tick ovaries are classified as panoistic ovaries with no nurse or follicle cells based on electron microscopic observations [27]. Our results showed there were small cells stained with *OmSpo* and these cells appear to play roles similar to the follicle or nurse cells in insect ovaries. Further functional analysis of the ovary, as shown in this study, greatly contributes to understanding the mechanisms regulating egg production in ticks.

In contrast to OmSpo, *OmShd* expression was observed in various tissues, especially the midgut (Fig 5). *OmShd* expression in various tissues may enable an organism to synthesize adequate amounts of 20E for the regulation of nymphal development and egg production. In insects, *Shd* expression is also detected in various tissues including midgut and fat bodies [26, 28–30]. However, the expression of *OmShd* in the fat bodies of *O*. *moubata* was faint. Tick fat bodies are a very fine tissue whereas the midgut is the largest tissue taking up most of the body cavity, so OmShd in the midgut may provide the highest activity for producing functional ecdysteroids.

### Differences in regulation of ecdysteroidogenic enzymes in nymphal and adult stages

In final instar nymphs, *OmSpo* and *OmShd* expression gradually increased after engorgement and peaked before molting (Fig 7). These increases correspond well with changes in ecdysteroid titers of the hemolymph [11] indicating increases in *OmSpo* and *OmShd* generate the appropriate changes in ecdysteroid titers to regulate molting of final instar nymphs. Because nymphs of *O*. *moubata* require engorgement for molting, nutrition appears to stimulate ecdysteroidogenesis.

Ecdysteroidogenesis in adult females is not simple; *OmShd* expression showed several surges from 1 day after engorgement prior to *OmSpo* expression (Fig 7). In mated adult females of *O*. *moubata*, ecdysteroid titers increase in the hemolymph immediately after engorgement with expression of receptors for egg production [11, 14, 31, 32]. Mated adult females show more frequent increases in *OmShd* than virgin adult females. As shown by dsShd injection, slight changes in *OmShd* expression may be sufficient to evoke visible changes in ticks. Therefore, the differences in *OmShd* expression between mated and virgin adult females appear to be critical for the production of functional ecdysteroid to complete egg production. The positive effect of mating on ecdysteroidogenesis has also been reported in females of *D*. *melanogaster* [33]. Therefore, a mechanism to stimulate ecdysteroidogenesis by mating may exist broadly in arthropods.

*OmSpo* expression in adult females peaked several days after engorgement (Fig 7). The amounts of ecdysteroids present in the ovaries gradually increased from 7 days after engorgement [11]. *OmSpo* expression appears to correspond to the change in ecdysteroids of the ovary, thus a key function of OmSpo in adult females may be the production of ecdysteroids stored in the oocytes. Ecdysteroids are accumulated in the oocytes as an inactivated form until embryonic development [34], so *OmShd* expression at the time of high *OmSpo* expression may not be necessary. Interestingly, *OmSpo* expression was similar in mated and virgin adult females (Fig 7), although virgin females show lower levels of ecdysteroids in the hemolymph [11]. This conflict indicates OmSpo is not the principal reason for the differences in ecdysteroid production between mated and virgin females. Further analyses, to clarify what leads to these interesting differences in ecdysteroidogenesis are necessary to understand the regulatory mechanisms that depend on mating.

## Conclusion

In this study, tick ecdysteroidogenesis was partially elucidated for the first time with *O*. *moubata* as the model acari. Processes regulated through Spook and Shade enzymes were shown to be conserved in *O*. *moubata*. Ticks appear to utilize the ovary or reproductive tissues for ecdysteroidogenesis without the establishment of separate specific steroidogenic tissues. In addition to mating, engorgement is an important factor for ecdysteroidogenesis because ecdysteroid synthesis and secretion are initiated after engorgement [11]. Obtaining cholesterol is necessary for ecdysteroid production. The nutrient signaling pathways, namely the target of rapamycin (TOR) pathway, is also related to ecdysteroidogenesis [35], so ecdysteroidogenesis in ticks may also be regulated by the TOR signaling pathway. Further analyses to evaluate the influence of mating and engorgement on ecdysteroid synthesis are required to fully understand the regulatory mechanisms of ecdysteroidogenesis in ticks.

## Supporting Information

S1 Fig Nucleotide sequences of *OmSpo*. Underlines indicate the region determined by RNA-seq.
(TIF)

S2 Fig Nucleotide sequences of *OmShd*. Underlines indicate the region determined by RNA-seq.
(TIF)

S3 Fig Effects of dsRNA injection. Relative expression levels of *OmSpo* in ovaries (A) and *OmShd* in midguts (B) after dsRNA injection. Abnormal exuvia after dsShd injection (C).
(TIF)

S4 Fig Ecdysteroids in the hemolymph of nymphs injected with dsRNA. Chromatograms of ecdysone and 20E in the hemolymph of nymphs determined by LC-MS/MS (A). Red line indicates 20E, while blue line indicates ecdysone. Intensity was indicated as counts per second (cps). MS/MS spectra of ecdysone and 20E detected in the hemolymph of nymphs injected with dsShd or standards (B). The amounts of ecdysone and 20E in the hemolymph of control or nymphs injected with dsShd determined using LC-MS/MS (C). u.d. indicates below detection limit.
(TIF)
