# Supplementary figures and images for: Ovarian Ecdysteroidogenesis in Both Immature and Mature Stages of an Acari, Ornithodoros moubata
Source: PLoS One. 2015 Apr 27;10(4):e0124953. doi: 10.1371/journal.pone.0124953 (PMC4411005; doi:10.1371/journal.pone.0124953)

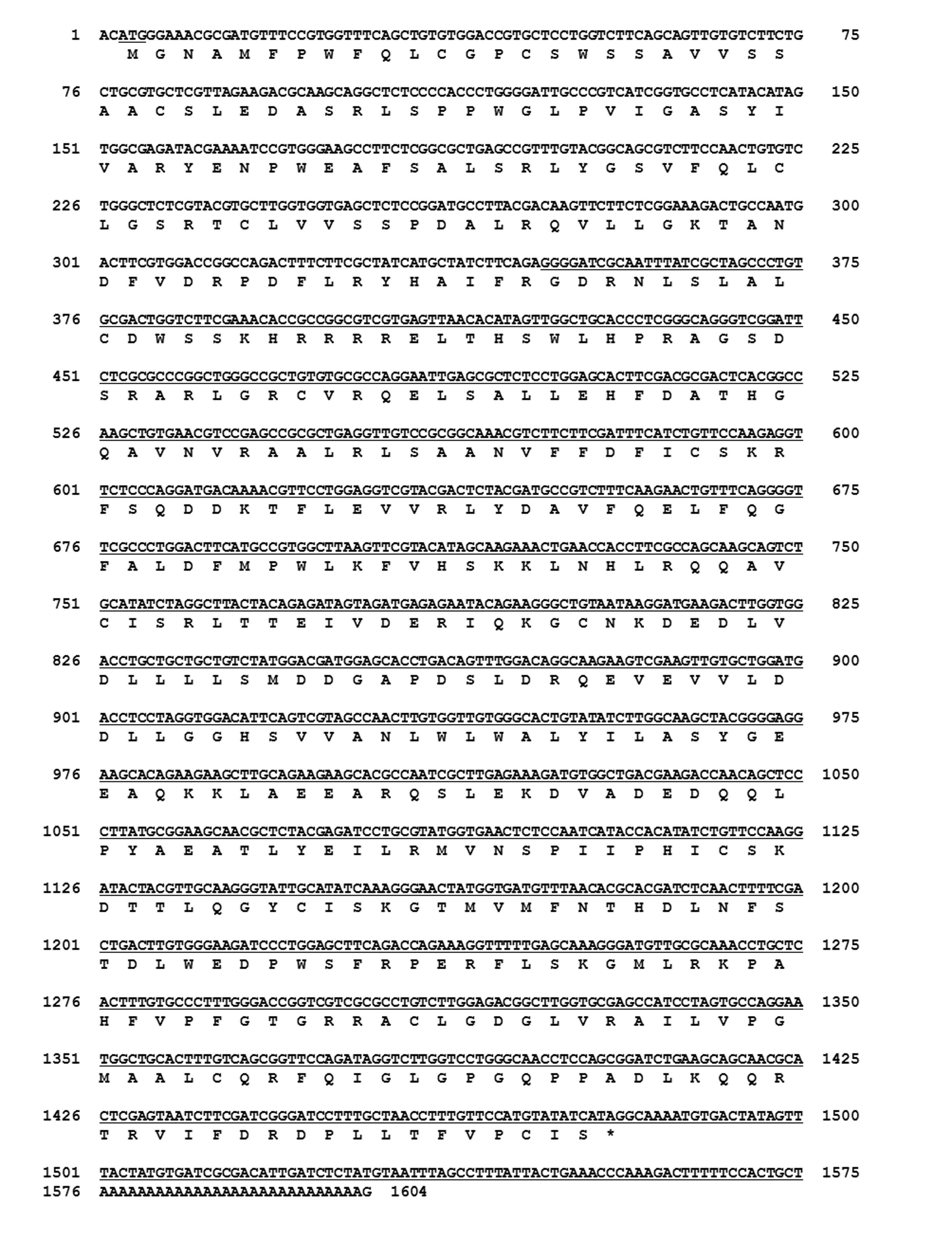

Supplement: S1 Fig — Underlines indicate the region determined by RNA-seq. (TIF) [file pone.0124953.s001.tif]

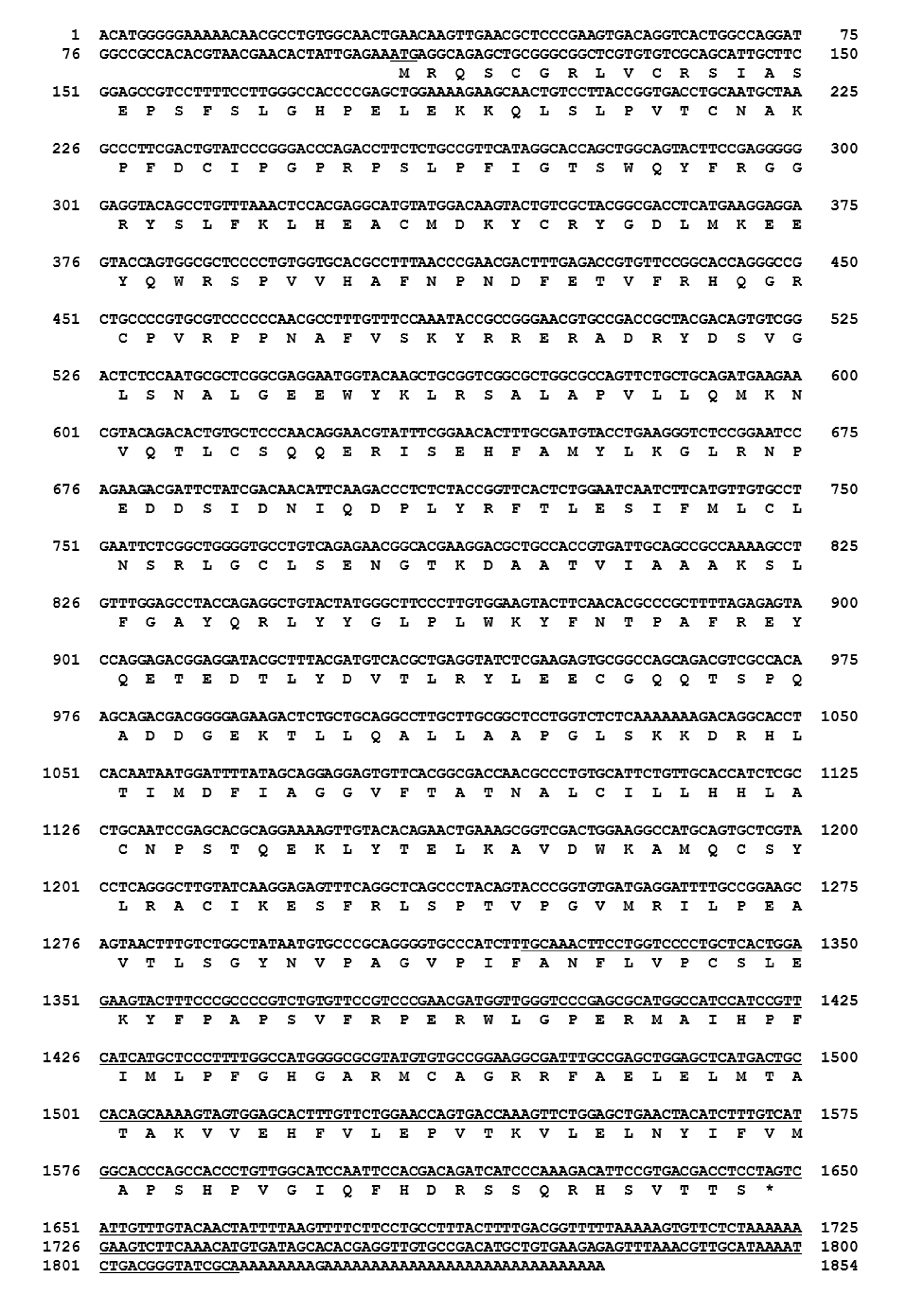

Supplement: S2 Fig — Underlines indicate the region determined by RNA-seq. (TIF) [file pone.0124953.s002.tif]

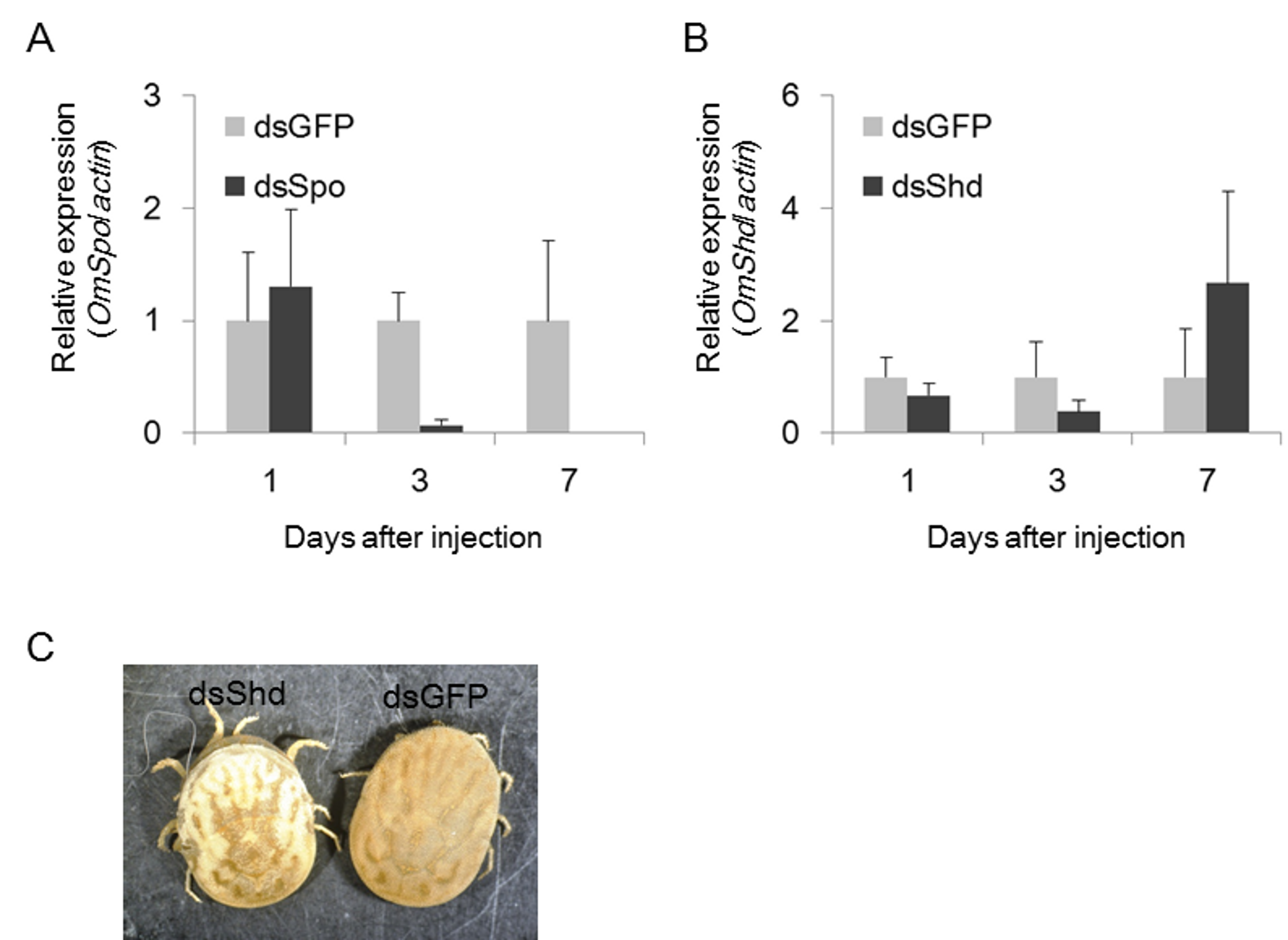

Supplement: S3 Fig — Relative expression levels of OmSpo in ovaries (A) and OmShd in midguts (B) after dsRNA injection. Abnormal exuvia after dsShd injection (C). (TIF) [file pone.0124953.s003.tif]

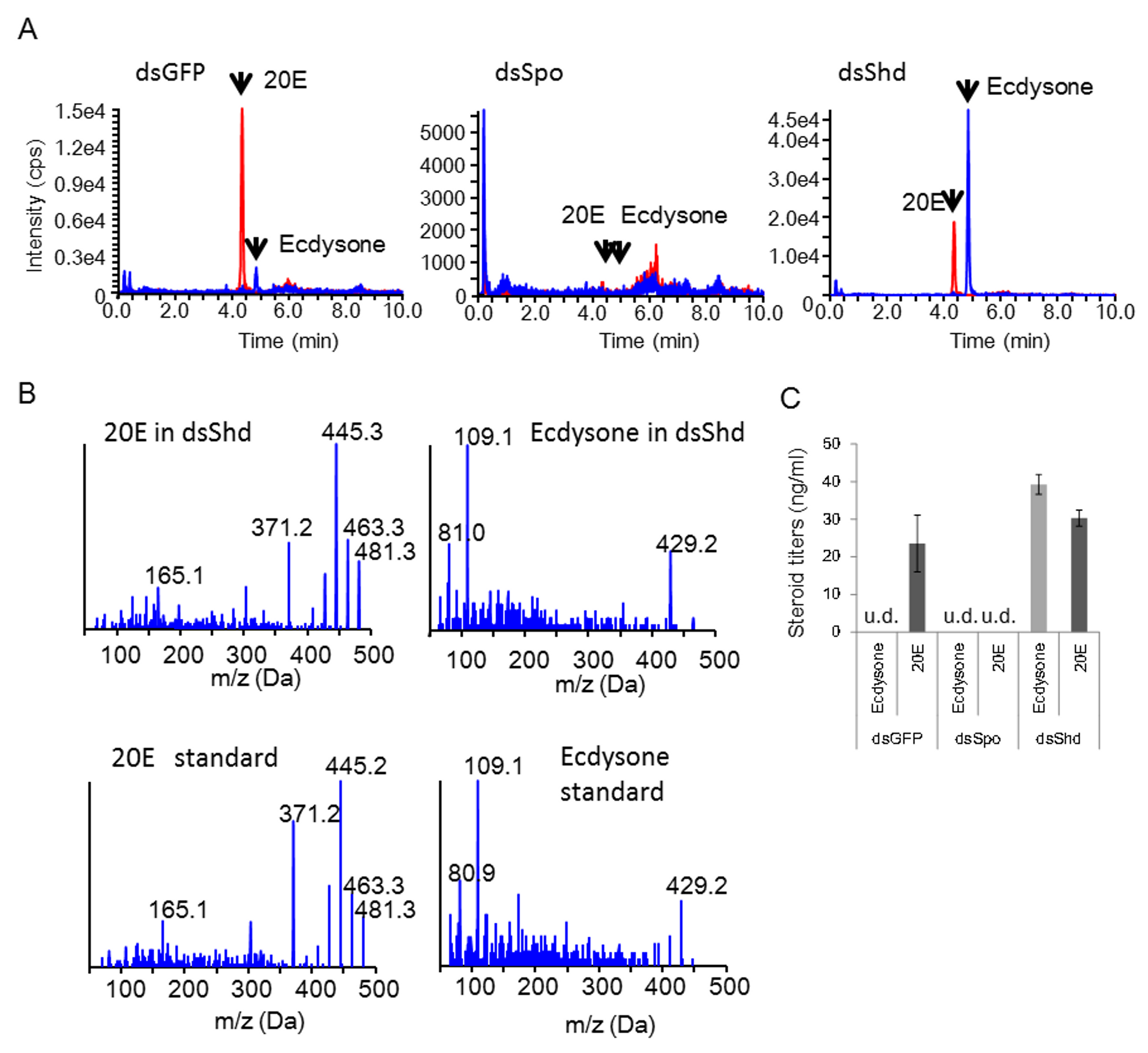

Supplement: S4 Fig — Chromatograms of ecdysone and 20E in the hemolymph of nymphs determined by LC-MS/MS (A). Red line indicates 20E, while blue line indicates ecdysone. Intensity was indicated as counts per second (cps). MS/MS spectra of ecdysone and 20E detected in the hemolymph of nymphs injected with dsShd or standards (B). The amounts of ecdysone and 20E in the hemolymph of control or nymphs injected with dsShd determined using LC-MS/MS (C). u.d. indicates below detection limit. (TIF) [file pone.0124953.s004.tif]
